# Isotretinoin-Associated Eruptive Milia: A Rare Adverse Effect

**DOI:** 10.7759/cureus.102725

**Published:** 2026-01-31

**Authors:** Fatima AlQaydi, Esmaeel AlMarzouqi

**Affiliations:** 1 Dermatology, Rashid Hospital, Dubai Health Authority, Dubai, ARE; 2 Dermatology, Mohammed Bin Rashid University of Medicine and Health Sciences, Dubai, ARE

**Keywords:** adverse effect, drug-induced skin reaction, eruptive milia, isotretinoin, retinoid

## Abstract

Isotretinoin is a widely used systemic retinoid for the treatment of moderate-to-severe acne vulgaris. While its common cutaneous side effects include dryness, cheilitis, and photosensitivity, eruptive milia is a rarely reported adverse event. Awareness of such uncommon associations is important for dermatologists managing patients on isotretinoin.

A 17-year-old male with moderate-to-severe acne vulgaris was initiated on oral isotretinoin at a dose of 30 mg daily. After two months of therapy, he developed multiple tiny, white, dome-shaped papules localized to the upper cheeks, mainly below the lower eyelids. Approximately 20 lesions were noted, clinically consistent with milia. There was no history of preceding trauma, cosmetic product use, or dermatologic procedures. Isotretinoin treatment was continued, and the milia were managed with simple manual extraction, leading to complete resolution without recurrence.

Milia are superficial keratin-filled cysts that typically arise in response to skin injury, inflammation, or certain topical agents. Although isotretinoin is known to modulate follicular keratinization, its role in the development of eruptive milia is not well understood. The temporal relationship in this case supports a possible drug-induced effect. Only a few similar cases have been reported in the literature, suggesting this may be an underrecognized side effect. Continuation of isotretinoin appears to be safe, and milia can be effectively managed with conservative extraction.

Eruptive milia may occur as a rare side effect of isotretinoin therapy. Recognizing this benign condition can help clinicians reassure patients and avoid unnecessary discontinuation of treatment.

## Introduction

Isotretinoin is a systemic retinoid indicated for the treatment of severe nodulocystic acne and moderate acne refractory to conventional therapies. Its therapeutic efficacy stems from reducing sebaceous gland size and sebum production, normalizing follicular keratinization, and exerting anti-inflammatory effects [1,2]. Despite its widespread use, isotretinoin is associated with several mucocutaneous adverse effects, including xerosis, cheilitis, epistaxis, and photosensitivity [3].

Milia are benign, keratin-filled epidermal inclusion cysts presenting as small, firm, white or yellowish papules, typically 1-2 mm in diameter. They commonly localize on the face, especially the periorbital region. Milia can be classified as primary or secondary, the latter often arising following cutaneous trauma, blistering disorders, cosmetic procedures, or certain medications [4].

Eruptive milia as a side effect of isotretinoin therapy is rare, with only limited cases documented in the literature [5]. The mechanism remains unclear but may be related to altered follicular keratinization induced by retinoids. We present a case of eruptive milia occurring in an adolescent male undergoing oral isotretinoin therapy for acne vulgaris, highlighting this uncommon but benign adverse reaction and its management without discontinuation of treatment.

## Case presentation

A 17-year-old male with no significant past medical or dermatologic history presented to our dermatology clinic with moderate inflammatory acne vulgaris predominantly affecting the face. The patient was started on oral isotretinoin therapy at a dose of 0.5 mg once daily, with no prior use of systemic retinoids or other acne treatments.

**Figure 1:**
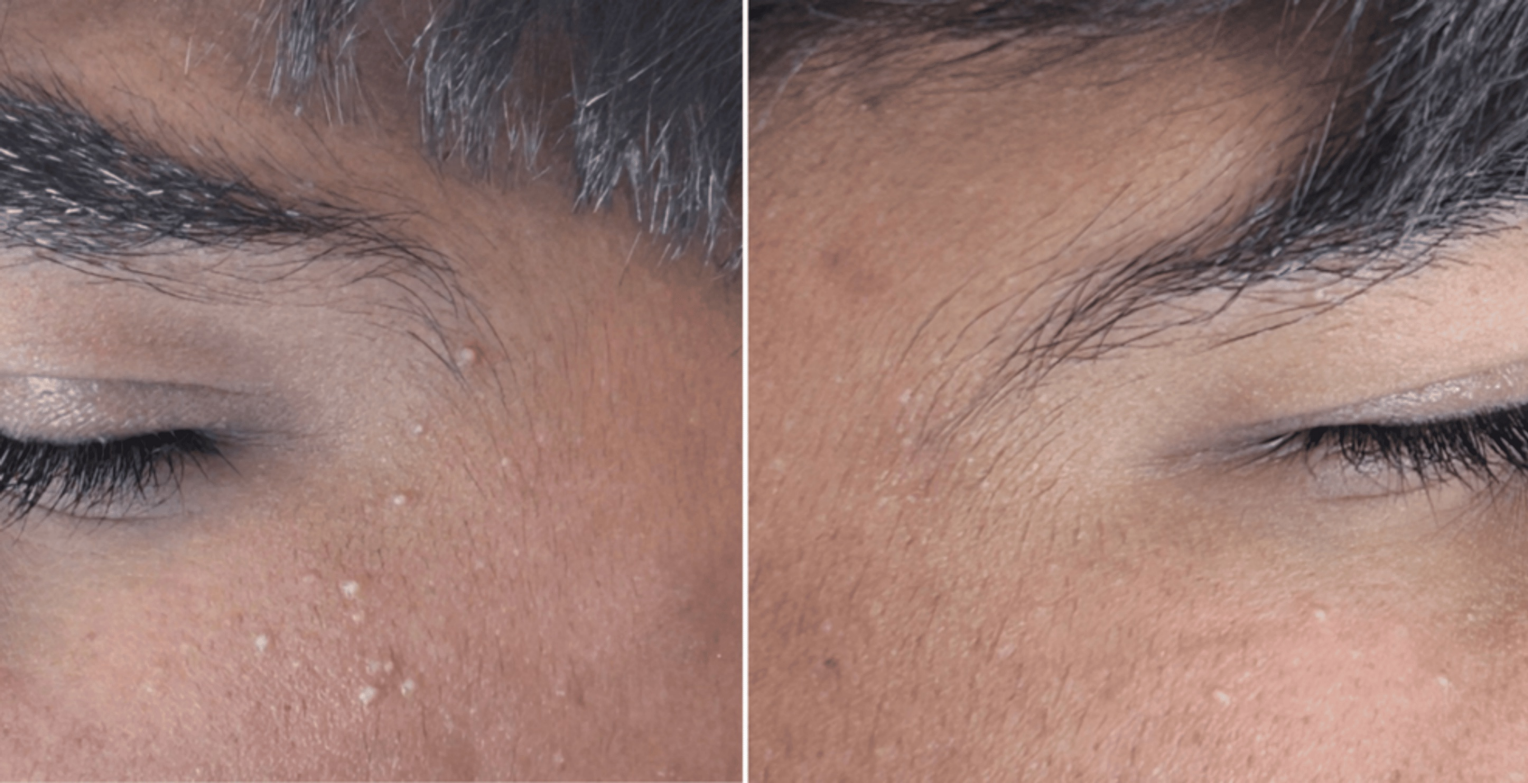
Multiple discrete, white, dome-shaped papules consistent with milia, distributed bilaterally over the temporal regions.

After approximately two months of continuous isotretinoin treatment, the patient demonstrated a marked improvement in his acne vulgaris, with a significant reduction in inflammatory papules and pustules and no new nodulocystic lesions. During this period, he developed multiple small, whitish papules on his face. Clinical examination revealed about 15 to 20 discrete, dome-shaped papules measuring 1-2 mm in diameter, distributed symmetrically on the upper cheeks, primarily concentrated below the lower eyelids bilaterally (Figure 1).

The lesions were asymptomatic and non-inflammatory, with no associated erythema, pustulation, or follicular plugging. Their uniform size, pearly-white appearance, and lack of inflammatory features distinguished them from comedonal or inflammatory acne lesions. Dermoscopic examination revealed well-defined, round, homogeneous white-to-yellowish structures without surrounding vascular patterns, findings consistent with milia and further supporting the diagnosis of eruptive milia.

**Figure 2:**
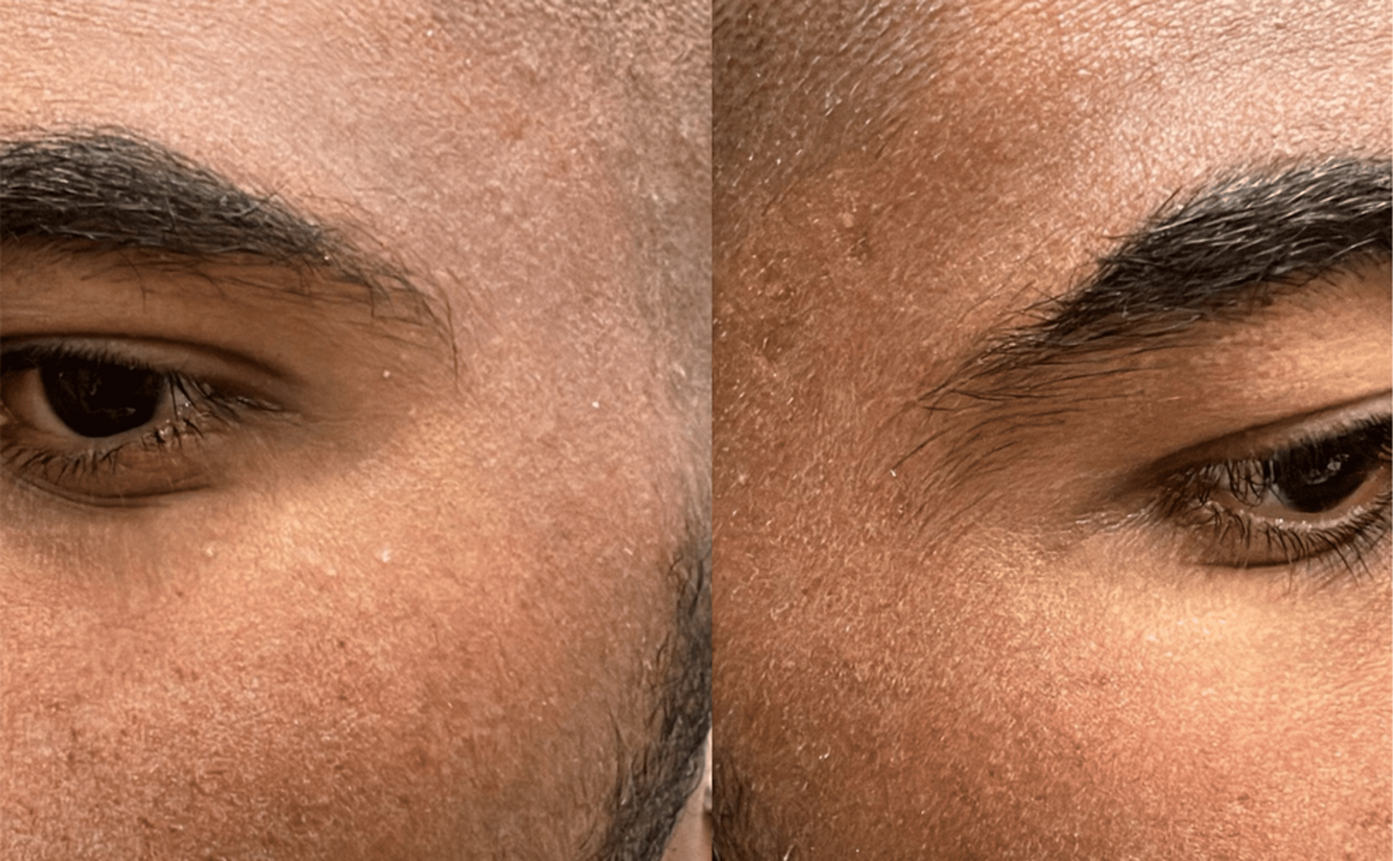
Follow-up picture showing resolution of milia after extraction.

Isotretinoin therapy was maintained at the same dosage without interruption. Management of the milia consisted of manual extraction during follow-up visits, which the patient tolerated well. The lesions resolved completely following extraction and represented a one-time eruption. No recurrence or development of new milia was observed after extraction (Figure 2), despite continuation of isotretinoin therapy throughout the follow-up period.

## Discussion

Eruptive milia as an adverse effect of isotretinoin therapy is exceedingly rare, with only a limited number of cases reported in the dermatologic literature [6]. This rarity may contribute to underrecognition among clinicians treating acne patients with systemic retinoids. Awareness of this benign but potentially distressing condition is essential to ensure proper management.

The pathogenesis of isotretinoin-associated eruptive milia remains incompletely understood. It is postulated that isotretinoin’s effects on follicular keratinization and epidermal differentiation may lead to keratin entrapment within the pilosebaceous units, resulting in the formation of keratin-filled cysts characteristic of milia [7,8]. Altered epidermal turnover and sebaceous gland suppression caused by isotretinoin may further disrupt normal follicular desquamation, thereby promoting cyst development [9]. Additionally, this presentation may reflect a paradoxical early retinoid-related reaction, analogous to the initial inflammatory “flare” or retinoid dermatitis sometimes observed at the onset of therapy. Although retinoid dermatitis typically manifests as erythema and scaling rather than milia, a similar transient disruption of epidermal differentiation and cutaneous barrier function may contribute to eruptive milia in susceptible individuals. These hypotheses align with the known pharmacodynamics of retinoids on cutaneous cell proliferation and differentiation.

Clinically, eruptive milia present as numerous small, firm, white to yellowish papules localized mainly to the facial region, often involving the periorbital area and cheeks [10]. Diagnosis is predominantly clinical, and biopsy is rarely required unless atypical features or diagnostic uncertainty exist. The main differential diagnoses include syringomas, sebaceous hyperplasia, and other epidermal cystic lesions, which may show overlapping clinical features. Syringomas typically present as multiple flesh-colored papules in the periorbital region, whereas sebaceous hyperplasia is characterized by soft papules with central umbilication and prominent vascular patterns. In this context, dermoscopy is a useful noninvasive adjunct, as milia characteristically appear as well-defined, homogeneous white to yellowish structures without associated vascular patterns, aiding in differentiation from other adnexal or epidermal lesions and reducing the need for biopsy.

Management typically involves conservative approaches, with manual extraction being the treatment of choice. Importantly, continuation of isotretinoin therapy is generally safe and does not appear to worsen or prolong the condition [6,11]. Thus, discontinuation of isotretinoin is not routinely indicated unless the lesions are symptomatic or significantly affect the patient’s quality of life.

## Conclusions

This case adds to the sparse literature on isotretinoin-induced eruptive milia and emphasizes that the condition is benign and manageable without cessation of essential acne therapy. Limitations include the absence of histopathologic confirmation, which was deemed unnecessary given the classic clinical presentation. Further studies with larger patient cohorts are needed to better characterize the incidence, pathophysiology, and optimal management strategies.

Clinicians should remain attentive to the possibility of eruptive milia in patients undergoing isotretinoin therapy and provide reassurance about its benign and manageable nature. Appropriate conservative management can effectively resolve the lesions, allowing uninterrupted continuation of isotretinoin therapy.
